# Combined resistance training with aerobic training improves physical performance in patients with coronary artery disease: A secondary analysis of a randomized controlled clinical trial

**DOI:** 10.3389/fcvm.2022.909385

**Published:** 2022-08-24

**Authors:** Tim Kambic, Nejc Šarabon, Mitja Lainscak, Vedran Hadžić

**Affiliations:** ^1^Cardiac Rehabilitation Unit, Department of Research and Education, General Hospital Murska Sobota, Rakičan, Slovenia; ^2^Faculty of Health Sciences, University of Primorska, Izola, Slovenia; ^3^Human Health Department, InnoRenew CoE, Izola, Slovenia; ^4^Laboratory for Motor Control and Motor Behaviour S2P, Science to Practice, Ltd., Ljubljana, Slovenia; ^5^Division of Cardiology, General Hospital Murska Sobota, Murska Sobota, Slovenia; ^6^Faculty of Medicine, University of Ljubljana, Ljubljana, Slovenia; ^7^Faculty of Sport, University of Ljubljana, Ljubljana, Slovenia

**Keywords:** strength training, cardiac rehabilitation, myocardial infarction, muscle strength, mobility, balance

## Abstract

**Background:**

The efficacy of combined resistance training (RT) and aerobic training (AT) compared with AT alone is well established in cardiac rehabilitation (CR); however, it remains to be elucidated whether RT load (high load [HL] vs. low load [LL]) modifies the outcomes. The aim of our study was to investigate the effects of HL-RT and LL-RT combined with AT in comparison to AT alone on body composition and physical performance in patients with coronary artery disease (CAD) enrolled in phase II CR.

**Methods:**

We randomized 79 patients with a stable CAD to 12 weeks of lower limb LL-RT + AT (35–40% of one repetition maximum [1-RM]; *n* = 28), HL-RT + AT (70–80% of 1-RM; *n* = 26), or AT (*n* = 25). Fifty-nine patients (75% men) with mean (standard deviation) age 61 (8) years and left ventricular ejection fraction 53 (9)% completed LL-RT (*n* = 19), HL-RT (*n* = 21) and AT (*n* = 19). Body composition and physical performance (upper and lower submaximal muscle strength, flexibility, balance, and mobility) were measured at baseline and post-training.

**Results:**

Training intervention had no significant impact on time × group interaction in the body composition measures. There was a significant time × group interaction for the gait speed test, chair sit-and-reach test, arm curl test, Stork balance test, up and go test, STS-5, and 6-min walk distance (*p*-values ≤ 0.001–0.04) following the training intervention. After the training intervention, HL-RT improved gait speed (+12%, *p* = 0.044), arm curl (+13%, *p* = 0.037), and time of Up and Go test (+9%, *p* < 0.001) to a greater extent compared with AT group, while there was a greater improvement in time of Up and Go test (+18%, *p* < 0.001) and time of five sit-to-stand tests (+14%, *p* = 0.016) following LL-RT when compared with AT. There were no differences between HL-RT and LL-RT in post-training improvement in any of the physical performance measures.

**Conclusion:**

The combination of AT with HL-RT or LL-RT promoted similar improvements in physical performance, which were superior to AT. Therefore, both types of combined AT and RT can be applied to patients with CAD.

**Clinical trial registration:**

[https://clinicaltrials.gov/ct2/show/NCT04638764] Identifier [NCT04638764].

## Introduction

Cardiac rehabilitation (CR) is recognized as a multidisciplinary intervention for secondary prevention and treatment of patients with coronary artery disease (CAD) (1–3), wherein exercise training presents a core component. Over the years, exercise training in CR has evolved from the single exercise modality intervention predominately based on aerobic training (AT) (4) to a multicomponent intervention consisting of AT and resistance training (RT), with flexibility, coordination, and balance training advised as adjunct training modalities (1).

In the past two decades, meta-analyses have shown the superior effects of combined AT with RT on maximal aerobic capacity (5, 6) and muscle strength (5–7) when compared with AT in patients with CAD. Despite being understudied in CR, high load (HL) RT has shown similar superiority as progressive low load (LL) to moderate load RT over AT alone (6). While the efficacy of combined RT and AT has been established, the dose-dependent relationship between RT load and improvement in physical performance remains to be established in patients with CAD. In healthy young and older adults, HL-RT (e.g., >70% of one repetition maximum [1-RM]) induced greater gains in maximal muscle strength (8, 9) and physical performance (8) compared with LL-RT (<40% of 1-RM) and had a similar effect on muscle hypertrophy (9). Therefore, we expect similar findings to be established also in patients with CAD.

To date, previous studies have reported changes in body composition (10–13) and physical performance (14) following combined AT and RT when compared with AT in patients with CAD. Despite conflicting results, some studies have shown an improvement in hip and waist circumference (14, 15), and body fat (10, 11) following both training modalities, while lean body mass increased only following combined AT and RT (11, 12). When comparing both training interventions, there were no differences in the waist and hip circumference (14, 15) and body fat mass (10), whereas only one study showed a greater increase in lean body mass following AT and RT in relation to AT (13). In addition, this study also showed a dose-dependent relationship between the weekly volume of RT (two vs. three weekly sessions) and improvement of lean leg mass following AT and RT compared with AT.

Furthermore, screening of physical performance following multicomponent exercise training has been limited in patients with CAD, despite a high prevalence of frailty and sarcopenia, especially among older patients (16). All available studies have shown improvement in flexibility (11, 15) in all training groups, while submaximal muscle strength improved only following combined AT and RT (7, 14). In addition, studies showed no between-group difference in flexibility (11, 15), and greater improvement in upper and lower limb submaximal muscle strength following combined AT and RT (13, 14). Moreover, it still must be established whether the combination of RT and AT provides additional benefits on submaximal endurance (e.g., 6-min walk test [6MWT]) compared with AT alone, which was previously associated with improved distance of 6MWT (17).

The aim of the study was to establish whether the dose-dependent relationship between RT load (HL- and LL-RT) and improvement in body composition and physical performance existed. Therefore, we hypothesized that HL-RT and LL-RT would elicit greater improvement of submaximal muscle strength and endurance, and greater decrease in body fat mass and an increase in lean body mass when compared with AT. In addition, we also hypothesized that HL-RT would provide greater gains in lower limb submaximal strength compared with LL-RT.

## Materials and methods

### Study design

In this randomized controlled clinical trial, patients with CAD were randomly assigned to three intervention groups using cluster randomization: HL-RT combined with interval AT; LL-RT combined with interval AT; and interval AT alone as standard care. The study was designed in accordance with Consolidated Standards of Reporting Trials (18) and the rationale and design of the study are available elsewhere (19). In line with standard procedures in CR, we used cluster randomization (sealed envelope for each randomized cluster) to allocate patients to each training group. We randomized between 3 and 5 patients to each cluster, depending on the Coronavirus-2019 hospital restrictions.

This study presents prespecified secondary outcomes of a randomized controlled clinical trial (19), while the primary outcomes were reported elsewhere (20). The outcomes of the study were: changes in physical performance (submaximal muscle strength, mobility, endurance, balance, and flexibility) and body composition (body mass, waist and hip circumference, lean and fat mass, and phase angle). The outcomes of the study were assessed by an experienced kinesiologist, which was not blinded to group allocation due to the protocols of routine CR during the Coronavirus-19 pandemic.

The measurements were conducted at baseline and after 36 training sessions. In addition to extensive baseline medical examination by a cardiologist and cardiopulmonary exercise test (CPET) performed on a separate day, all other outcomes of the study were assessed during a single visit to the out-patient CR center. On the measurement day, we first measured patients‘ body composition, followed by flexibility tests, mobility test, upper and lower limb muscle strength test, familiarization of patients with RT on the leg press machine, and finished with a submaximal endurance test in a fatigue minimized order.

### Participants

We recruited patients with a stable CAD (after acute coronary syndrome and/or percutaneous coronary intervention) from the Division of Cardiology, General Hospital Murska Sobota, Slovenia. Only patients 18–85 years old, with left ventricular ejection fraction ≥ 40%, documented CAD (acute coronary syndrome and/or percutaneous coronary intervention about 1 month after event or procedure), referred to phase II CR and with the completed baseline cardiopulmonary exercise test were included in the study (1). Exclusion criteria were in accordance with the previous recommendation and are available elsewhere (17, 19). Recruitment of patients started in July 2020 and was completed in June 2021.

### Training protocol

Patients performed three exercise sessions per week for 12 weeks or until the completion of 36 sessions), with at least 48 h rest between sessions. Each training session consisted of three periods: general warm-up (10 min dynamic flexibility exercises followed by calisthenics using elastic bands and/or LL dumbbells and balance exercises), main period (45 min of AT and 10 min of RT), and cool-down period (5 min static stretching and breathing exercises). After a general warm-up, patients in all three groups performed aerobic interval cycling (3–5 min of workload cycling separated by 2 min of rest) progressing from 50% of maximal peak power achieved at baseline CPET to 80% of peak power (19).

In addition, patients in both RT groups performed a total of 36 sessions on a leg press machine (three measurements of 1-RM and 33 RT sessions). Both RT groups differed in training load, whereas the training volume was matched by the number of repetitions. The range of repetitions and progression of RT followed previously published recommendations (1, 17, 21, 22). The workload in the HL-RT group progressed from the initial three sets at an intensity of 70% of 1-RM (6–11 repetitions per set) to 80% of 1-RM (6–8 repetitions per set). The workload in the LL-RT group progressed from the initial three sets at an intensity of 35% of 1-RM (12–22 repetitions per set) to 40% of 1-RM (12–16 repetitions per set). During the eighth week of training (on 22nd training session), patients’ 1-RM was re-evaluated in all three groups and the new 1-RM was used to prescribe RT for the final part of the intervention. During the final 4 weeks of training, the load in the HL-RT group was progressed from 70% 1-RM (11 repetitions per set) to 80% 1-RM (6–8 repetitions per set), and the load the in LL-RT group progressed from 35% 1-RM (22 repetitions per set) to 40% 1-RM (12–16 repetitions per set). A lifting cadence of 1 s: 1 s (concentric and eccentric contraction) was used, with 90 s rest between sets (23). Patients were familiarized with proper lifting techniques and were instructed to inhale during the eccentric contraction and exhale during the concentric contraction to avoid the Valsalva maneuver (17, 21).

Patients’ safety was ensured with continuous monitoring of heart rate and blood pressure before, during, and after each training modality. The intervention was guided by a kinesiologist, while safety was ensured by a medical nurse and physiotherapist, with a cardiologist available for consultations on-site. Additional safety procedures are in detail described elsewhere (19).

### Measurements

#### Cardiopulmonary exercise test

Maximal aerobic capacity was assessed using an adjusted ramp protocol (24) on a Schiller ERR 911 ergometer and a Cardiovit CS-200 excellence ergo-spirometer (Schiller, Baar, Switzerland) to determine workload for AT. After short instruction, patients completed a spirometry test. Afterward, we determined patients‘ baseline heart rate, blood pressure, and gas exchange in a seated position. The test was initiated with patients who started cycling without workload for 3 min at 50–60 rpm, followed by increasing workload every minute for an additional 10–25 W until exhaustion and/or exercise-limiting contraindications. The test was conducted by a kinesiologist and supervised by a medical nurse.

#### Measurements of body composition

Body height and mass were measured on Marsden DP3810 weighing scale and stadiometer (Marsden Weighing Group, Rotherham, United Kingdom); and waist and hip circumference were measured with a standard measuring tape. Waist circumference was measured midpoint between lowest rib and the iliac crest, and the hip circumference was measured in the widest part of the gluteal region. The average circumference out of two measurements was used in the final analysis (14).

Body composition was assessed in the morning (before 10 a.m.) using bioimpedance measurement with a Bodystat Quadscan 4000 Touch (Bodystat, Douglas, Isle of Man, United Kingdom) with patients lying in a supine position after 10 min rest. Electrodes were connected to the hands (wrist and middle finger) and feet (ankle and above the knuckle of the toe) after those areas were cleaned with alcohol. Patients were asked to be fasted and to report their fluid intake prior to measurement. Post-training assessment of body composition was performed during the same time of the day as during the baseline assessment (±2 h). Outcome measures were body fat, lean body mass, and phase angle (ratio between cellular [intracellular and extracellular] resistance and membrane-specific reactance) as a marker of cellular health (25). All body composition variables were calculated according to the manufacturer’s guidelines using height, mass, waist and hip circumference, age, and sex data.

#### Measurement of maximal leg strength

Patients underwent leg-press familiarization and performed submaximal strength tests using a Life Fitness Leg Press Pro 2 (Life Fitness Inc., Rosemont, IL, United States). Following standard warm-up (see section “Training protocol”), patients were familiarized with proper lifting techniques and leg press testing. During the measurement, patients performed a specific warm-up comprising of eight and six repetitions at 50 and 70% of their perceived 1-RM, respectively. After short rest, the weight was progressively increased until reaching the workload that can be lifted three to five times (3–5 RM). There was a 2–3 min rest between the trials (22). The 1-RM was calculated using the established 1-RM prediction equation (predicted 1-RM = maximal load lifted/1.0278–0.0278 × number of repetitions) (26).

#### Measurement of physical performance

Patients underwent extensive evaluation of physical performance to evaluate mobility, submaximal muscle strength, upper and lower limb flexibility, postural balance, and submaximal endurance, using a 4 m spontaneous gait speed test, hand grip test, arm curl test, five repetition sit-to-stand test (STS-5), back scratch test, chair sit-and-reach test, up and go test, Stork balance test, and 6MWT distance (27, 28).

##### Mobility, flexibility, gait speed, and balance

The usual-pace gait speed test was conducted on a 4 m track, and the best time of two measurements was used for further calculation of speed (28).

The Up and Go test was performed two times with 60 s of break between trials on 8 feet track (2.44 m), and the time required to get up from a seated position, walking, and turning at 2.44 m, and returning to a seated position was used in the final analysis (27).

The back scratch test and the chair sit-and-reach test assessed the flexibility of upper limb and lower limbs, respectively (27). In the back scratch test, the distance between fingers was measured two times, and the nearest distance was used as an outcome (negative value was marked when middle fingers could not touch each other, otherwise the result presented a positive value).

In the chair sit-and-reach test, the distance between the extended tip of middle finger and the tip of toe in the forward bend sitting position was measured two times on each leg, and the nearest distance was used in the final analysis (positive value = middle finger reached over the tip of toe, otherwise the result presented a negative value) (27).

The Stork balance test was measured while patients maintained the position on one leg with their hips on the hips and the other foot against the medial side of the knee of the stance leg. The best time (in seconds) out of three trials was used in the final analysis (29).

##### Submaximal muscle strength and endurance

The hand grip strength test was assessed using the Jamar Smart Hand dynamometer (Patterson Medical Ltd., Warrenville, IL, United States). Patients performed three repetitions, with 60 s of rest in a sitting position with an elbow flexed at a 90° angle. The highest value expressed in kg was included as an outcome (30).

The upper limb strength-endurance was assessed using the arm curl test, and patients were instructed to perform a maximal number of elbow flexions in 30 s. A dumbbell weight of 2.27 and 3.63 kg was used for women for men, respectively (27).

STS-5 estimated lower limb muscle strength and was performed two times, with a 60 s of rest. The fastest time of the fifth stand was used as an outcome (28).

The one-leg heel raise test was performed until exhaustion on a step wearing sports shoes (without heel), with fingertip touching the wall at shoulder height, knee fully extended, and the non-dominant leg held above the floor. A cadence of 60 heel raise cycles/min was used and the maximal number of correctly repeated heel raises was used as an outcome (31).

The 6-min walk test (6MWT) was performed on a 30 m track, and the total distance measured to the nearest meter was included as an outcome. Before and after the test, we measured heart rate, blood oxygen saturation, and blood pressure, and patients reported Borg dyspnea score on a 0–10 points scale (32, 33).

### Statistical analysis

The initial sample size calculations were based on mean changes (post-training – baseline values) in aerobic capacity or isometric maximal knee-extensor torque (primary outcomes) when comparing the effects of combined AT with either HL-RT or LL-RT with standard care (AT), and the exact calculations are available elsewhere (19, 20). Since we prespecified the analysis of the secondary outcomes in our study protocol (19), we also performed sample size calculations for the present sub-analysis. The calculations were based on previously reported mean changes in the Up and Go test when comparing the effect of HL-RT with LL-RT in older healthy adults. Calculations showed that at least 15 patients with CAD (5 patients per group) needed to be enrolled to detect a mean change of 0.6 s in the up and go test, assuming a statistical power of 0.90 (β = 0.10) and α = 0.05 (34).

Categorical variables are presented as numbers and percentages, and numerical variables are presented as means and standard deviations or median and interquartile (asymmetrically distributed variables). We screened all numeric variables for the normality of distribution (Shapiro–Wilk test), and homogeneity of variances (Levene’s test). The final analysis was performed according to per-protocol analysis, including all patients with completed 24–36 sessions (19). We assessed between-group differences in baseline and post-training change (% change = post-training value - baseline value/baseline value × 100%) using the one-way analysis of variance (ANOVA) or Kruskal–Wallis test (for asymmetrically distributed variables). In addition to both tests, we performed *post hoc* analysis using the Tukey or Bonferroni test. The effect of training intervention was calculated using unbalanced group two-way repeated measures ANOVA or analysis of covariance (ANCOVA), when analysis of baseline measures indicated a significant between-group difference. We used Bonferroni adjustment within two-way ANOVA to calculate the within-group effects of training intervention. In addition to ANCOVA, we assessed the within-group improvement following training intervention using the paired sample *t*-test or the Wilcoxon test, where necessary. Partial eta squared was used to calculate the effect size of each variable. The analysis was conducted using IBM SPSS 25 software (SPSS Inc., Armonk, NY, United States) at the level of statistical significance set at alpha < 0.05.

## Results

**FIGURE 1:**
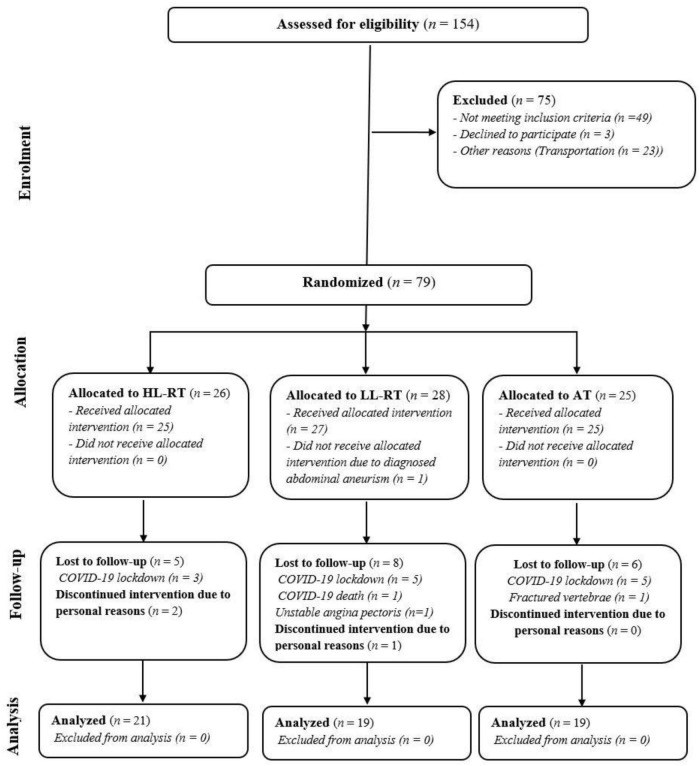
CONSORT flowchart of the study. HL-RT, high load-resistance training; LL-RT, low load-resistance training; CON, aerobic training; COVID-19, Coronavirus-19.

We screened 154 patients with CAD and included 79 patients in the study (Figure 1). Fifty-nine patients were included in the final analysis, age = 61 (8) years, height = 172.1 (8.4) cm, weight = 85.47 (15.43) kg, and left ventricular ejection fraction = 53 (9)%. Patients were enrolled 2 (1.5–3.0) months post-acute coronary syndrome or percutaneous coronary intervention in the study. At baseline, there were no between-group differences in baseline body height and weight, clinical characteristics, smoking status, and pharmacological therapy (Table 1). Atrial fibrillation was more prevalent in the AT group than in HL-RT and LL-RT groups (*p* = 0.038).

**TABLE 1:** Baseline anthropometry, clinical characteristics, and cardiovascular risk factors.

	Sample (*n* = 59)	AT group (*n* = 19)	LL-RT group (*n* = 19)	HL-RT group (*n* = 21)	*P* (ANOVA)
Age (years)	61 (8)	61 (9)	61 (7)	62 (8)	0.910
Gender [males, (%)]	44 (75)	14 (74)	15 (79)	15 (71)	0.931

**Anthropometry**					

Height (cm)	172.1 (8.4)	170.4 (8.8)	172.8 (8.6)	172.9 (7.9)	0.582
Weight (kg)	85.47 (15.43)	90.94 (19.04)	81.46 (13.37)	84.15 (12.56)	0.148

**Clinical data**					

LVEF (%)	53 (9)	50 (45, 60)	55 (50, 60)	50 (45, 58)	0.454
Time from clinical event to inclusion to CR (months)	2.0 (1.5, 3.0)	2.0 (2.0, 2.5)	2.5 (1.5, 3.0)	2.0 (1.5, 2.8)	0.832

**Myocardial infarction, *f* (%)**					

NSTEMI	25 (42)	9 (47)	8 (42)	8 (38)	
STEMI	24 (41)	7 (37)	7 (37)	10 (48)	0.947
Unstable AP/PCI	10 (17)	3 (16)	4 (21)	3 (14)	

**Comorbidities and risk factors**					

Arterial hypertension	41 (70)	15 (79)	11 (58)	15 (71)	0.383
Hyperlipidaemia	49 (83)	16 (84)	14 (74)	19 (91)	0.384
Diabetes	9 (15)	4 (21)	3 (16)	2 (10)	0.602
Atrial fibrillation	5 (9)	4 (21)	1 (5)	0 (0)	0.038
Thyroid disease	5 (9)	2 (11)	2 (11)	1 (5)	0.727
Renal disease	4 (7)	0 (0)	2 (11)	2 (10)	0.534

**Smoking, *f* (%)**					

non-smoker	14 (24)	3 (16)	3 (16)	8 (38)	
ex-smoker	35 (59)	13 (68)	11 (58)	11 (52)	0.346
smoker	10 (17)	3 (16)	5 (26)	2 (10)	

**Pharmacological therapy, *f* (%)**					

Aspirin	57 (97)	17 (90)	19 (100)	21 (100)	0.200
Beta blocker	59 (100)	19 (100)	19 (100)	21 (100)	1.000
ACE inhibitor/ARB	58 (98)	19 (100)	18 (95)	21 (100)	0.644
Statin	59 (100)	19 (100)	19 (100)	21 (100)	1.000
Antiplatelet drug	58 (98)	18 (95)	19 (100)	21 (100)	0.644
Anticoagulation drug	5 (9)	3 (16)	1 (5)	1 (5)	0.509
Diuretic	5 (9)	4 (21)	0 (0)	1 (5)	0.071

Data are presented as mean (standard deviation) or as median (first quartile, third quartile), AT, aerobic training; LL-RT, low load resistance training; HL-RT, high load resistance training; LVEF, left ventricular ejection fraction; (N)STEMI, (non-) ST segment-elevated myocardial infarction; AP, angina pectoris; PCI, percutaneous coronary intervention; ACE, angiotensin-converting enzyme; ARB, angiotensin II receptor blockers.

All measurements and the training intervention were performed without major cardiovascular events or complications (e.g., angina pectoris, blood pressure > 220/110 mmHg, palpitation, atrial fibrillation, arrhythmias, etc.). With the exception of some reports of the delay onset of muscle soreness following the baseline heel raises test and the 1-RM test, no exercise-limiting musculoskeletal problems were noted.

All patients completed all 36 sessions to out-patient CR apart from two patients in the HL-RT group with 24 completed sessions. Adherence to AT (one patient completed 35 sessions in AT group; one patient completed 34 sessions; and four patients completed 35 sessions in the LL-RE group; two patients completed 35 sessions in the HL-RT group) and RT (one patient completed 35 sessions) was high, with no between-group difference in adherence to AT (*p* = 0.240) and RT (*p* = 0.475).

Table 2 presents the change in body composition following training intervention in all groups. At baseline, there was a significant difference between groups in hip circumference (LL-RT vs. AT = −7.7 cm, *p* = 0.017) and fat mass (LL-RT vs. AT = −8.20 kg, *p* = 0.035). When adjusted for baseline difference, there were no significant differences between groups in post-training hip circumference and fat mass. Two-way repeated measures ANOVA has demonstrated a significant time effect for waist circumference, hip circumference, waist to hip ratio, fat mass, and lean mass (*p*-values = < 0.001–0.002, η^2^ = 0.166–0.896), and no significant time × group interaction for none of the body composition variables. There was a decrease in waist circumference in the LL-RT group (−2.5 cm, *p* = 0.001) and borderline decrease in HL-RT (−1.4 cm, *p* = 0.056) following the training intervention. AT group significantly increased fat% (+1%, *p* = 0.048), decreased lean mass% (−1%, *p* = 0.048), and lean mass (−1.05 kg, *p* = 0.016) following the training intervention. When comparing the groups in post-training improvement (% change), there was no significant difference in any of the body composition variables.

**TABLE 2:** Body composition at baseline and post-training.

							2-way ANOVA/ANCOVA	
		*N*	Baseline	Post-training	% change	*P* (% change)	Time effect/Effect of baseline	Interaction/Post-training difference
Weight (kg)	AT	19	90.94 (19.04)	90.49 (17.87)	0 (4)		*p* = 0.187	*p* = 0.974
	LL-RT	19	81.46 (13.37)	80.91 (13.90)	−1 (4)	0.775	η^2^ = 0.031	η^2^ = 0.001
	HL-RT	21	84.15 (12.56)	83.47 (13.48)	−1 (3)			

Waist (cm)	AT	19	108.9 (14.5)	107.7 (13.6)	−0 (−3, 1)		*p* < 0.001	*p* = 0.381
							η^2^ = 0.216	η^2^ = 0.034
	LL-RT	19	99.9 (10.2)	97.3 (10.4)	−3 (−5, 0)	0.140		
	HL-RT	21	101.7 (10.1)	100.3 (10.5)	−2 (−2, −0)			

Hip (cm)	AT	19	109.6 (10.1)	109.4 (8.6)	−1 (−2, 1)		***p* < 0.001**	***p* = 0.370**
	LL-RT	19	101.9 (5.1)	101.5 (5.5)	−2 (−4, 0)	0.611	**η^2^ = 0.896**	**η ^2^ = 0.036**
	HL-RT	21	104.8 (6.5)	104.4 (6.5)	−2 (−2, 0)			

WHR	AT	19	0.99 (0.07)	0.98 (0.08)	0 (−2, 2)		*p* < 0.001	*p* = 0.155
	LL-RT	19	0.98 (0.07)	0.96 (0.07)	0 (−2, 0)	0.079	η^2^ = 0.254	η^2^ = 0.064
	HL-RT	21	0.97 (0.06)	0.96 (0.06)	−1 (−1, 1)			

Fat (%)	AT	19	28.2 (9.2)	29.2 (8.7)	5 (8)		*p* = 0.500	*p* = 0.138
	LL-RT	19	22.3 (4.7)	22.0 (5.2)	−2 (11)	0.104	η^2^ = 0.008	η^2^ = 0.070

	HL-RT	20	24.9 (8.4)	24.7 (7.6)	1 (9)			
Fat (kg)	AT	19	26.0 (11.0)	26.7 (10.3)	5 (12)		***p* < 0.001**	***p* = 0.095**
							**η^2^ = 0.900**	**η^2^ = 0.083**
	LL-RT	19	17.8 (3.3)	17.6 (4.5)	−2 (14)	0.157		
	HL-RT	20	21.0 (8.3)	20.5 (7.5)	−1 (11)			

Lean (%)	AT	19	71.8 (9.2)	70.8 (8.7)	−2 (−3, 1)		*p* = 0.497	*p* = 0.139
	LL-RT	19	77.7 (4.7)	78.0 (5.2)	1 (−2, 3)	0.086	η^2^ = 0.008	η^2^ = 0.069
	HL-RT	20	75.1 (8.4)	75.3 (7.6)	0 (−2, 3)			

Lean (kg)	AT	19	64.9 (13.6)	63.9 (13.1)	−2 (3)		*p* = 0.002	*p* = 0.354
	LL-RT	19	63.6 (12.5)	63.3 (12.4)	0 (2)	0.372	η^2^ = 0.166	η^2^ = 0.037
	HL-RT	20	63.4 (11.7)	62.4 (11.5)	−2 (3)			

Dry lean (kg)	AT	19	14.3 (4.1)	14.3 (4.0)	1 (3)		*p* = 0.168	*p* = 0.322
	LL-RT	19	14.2 (3.9)	14.0 (3.9)	−1 (4)	0.181	η^2^ = 0.034	η^2^ = 0.040
	HL-RT	20	14.3 (4.2)	14.1 (4.3)	−2 (5)			

Phase angle (°)	AT	19	6.5 (1.0)	6.5 (0.7)	2 (8)		*p* = 0.138	*p* = 0.755
	LL-RT	19	6.8 (0.9)	6.8 (1.0)	1 (5)	0.663	η^2^ = 0.040	η^2^ = 0.010
	HL-RT	20	6.4 (0.8)	6.5 (0.9)	2 (7)			

Data are presented as mean (standard deviation) or as median (first quartile, third quartile). WHR, waist to hip ratio, LL-RT, low load resistance training, HL-RT, high load resistance training, AT, aerobic training, ANOVA, analysis of variance, ANCOVA, analysis of covariance, and η^2^, partial eta squared (effect size). Text in bold presents ANCOVA results.

All training groups had a similar level of baseline physical performance (Table 3), with the exception of the heel raise test (LL-RT vs. AT = + 8 repetitions, *p* = 0.012). When adjusted for baseline difference, AT group performed significantly less heel raises compared with the LL-RT group (adjusted mean difference = 6 repetitions, *p* = 0.022) and HL-RT group (adjusted mean difference = 7 repetitions, *p* = 0.002) following the training intervention. Two-way repeated measures ANOVA has demonstrated a significant time effect for all physical performance tests (all *p* < 0.001, η^2^ = 0.235–0.708), and a significant time × group interaction for the gait speed test, chair sit-and-reach test, arm curl test, Stork balance test, up and go test, STS-5 and 6MWT distance (*p*-values = < 0.001–0.04, η^2^ = 0.108–0.350).

**TABLE 3:** Physical performance at baseline and post-training.

							2-way ANOVA/ANCOVA	
		*N*	Baseline	Post-training	% change	*P* (%change)	Time effect/Effect of baseline	Interaction/Post-training difference
Up and Go test	AT	19	4.90 (0.79)	4.74 (0.76)	−7 (−10, 7)		*p* < 0.001	*p* < 0.001
	LL-RT	19	5.43 (1.36)	4.08 (0.81)	−25 (−33, −20)	0.000	η^2^ = 0.634	η^2^ = 0.350
	HL-RT	21	5.18 (0.95)	4.27 (0.70)	−16 (−25, −9)			

Gait speed (m/s)	AT	19	1.32 (0.26)	1.37 (0.26)	2 (−6, 14)		*p* < 0.001	*p* = 0.035
	LL-RT	19	1.34 (0.19)	1.53 (0.21)	9 (1, 32)	0.041	η^2^ = 0.397	η^2^ = 0.113
	HL-RT	21	1.31 (0.17)	1.50 (0.20)	14 (3, 23)			

Stork balance test (s)	AT	19	59.25 (49.44)	81.46 (62.34)	32 (7, 138)		*p* < 0.001	*p* = 0.018
	LL-RT	19	67.79 (47.24)	116.56 (69.69)	62 (34, 119)	0.213	η^2^ = 0.588	η^2^ = 0.134
	HL-RT	21	59.93 (44.74)	113.19 (75.34)	77 (42, 164)			

Back scratch test (cm)	AT	19	−13 (15)	−11 (12)	Na		*p* < 0.001	*p* = 0.668
	LL-RT	19	−10 (10)	−6 (11)	Na	Na	η^2^ = 0.235	η^2^ = 0.014
	HL-RT	21	−10 (12)	−7 (13)	Na			

Chair Sit-and-reach test (cm)	AT	19	2 (11)	5 (9)	Na		*p* < 0.001	*p* = 0.020
	LL-RT	19	0 (13)	7 (12)	Na	Na	η^2^ = 0.545	η^2^ = 0.130
	HL-RT	21	−3 (12)	4 (11)	Na			

Sit-and-Reach test (cm)	AT	19	14 (11)	16 (11)	Na		*p* < 0.001	*p* = 0.083
	LL-RT	19	15 (11)	19 (11)	Na	Na	η^2^ = 0.475	η^2^ = 0.085
	HL-RT	21	10 (13)	15 (10)	Na			

Hand grip strength (kg)	AT	19	42.2 (9.7)	44.3 (10.2)	6 (2, 13)		*p* < 0.001	*p* = 0.397
	LL-RT	19	43.4 (12.0)	47.2 (10.2)	7 (−1, 24)	0.886	η^2^ = 0.311	η^2^ = 0.034
	HL-RT	21	42.5 (11.8)	46.8 (11.5)	8 (−1, 12)			

Arm curl test (reps)	AT	19	22 (6)	24 (6)	10 (15)		*p* < 0.001	*p* = 0.009
	LL-RT	19	25 (6)	29 (4)	18 (18)	0.039	η^2^ = 0.605	η^2^ = 0.154
	HL-RT	21	22 (6)	27 (6)	23 (13)			

STS-5 test (s)	AT	19	8.55 (1.30)	7.77 (1.04)	−9 (10)		*p* < 0.001	*p* = 0.020
	LL-RT	19	8.73 (2.25)	6.54 (1.10)	−20 (16)	0.012	η^2^ = 0.517	η^2^ = 0.130
	HL-RT	21	8.35 (2.13)	6.79 (1.45)	−16 (13)			

Heel raise test (reps)	AT	19	16 (7)	20 (8)	25 (6, 36)		*p* < 0.001	*p* = 0.002
	LL-RT	19	24 (10)	32 (8)	26 (16, 45)	0.056	η^2^ = 0.598	η^2^ = 0.209
	HL-RT	21	19 (7)	29 (10)	36 (25, 72)			

6-MWT (m)	AT	19	508 (89)	554 (84)	10 (1, 15)		*p* < 0.001	*p* = 0.041
	LL-RT	19	531 (90)	613 (79)	15 (8, 22)	0.158	η^2^ = 0.708	η^2^ = 0.108
	HL-RT	21	523 (83)	592 (86)	12 (7, 19)			

Data are presented as mean (standard deviation) or as median (first quartile, third quartile). STS, sit-to-stand test five times; 6MWT, 6-min walk test; LL-RT, low load resistance training; HL-RT, high load resistance training; AT, aerobic training; Na, not applicable; ANOVA, analysis of variance; ANCOVA, analysis of covariance, and η^2^-partial eta squared (effect size). Text in bold presents ANCOVA results.

HL-RT and LL-RT significantly improved gait speed (both *p* < 0.001), upper limb flexibility (LL-RT, *p* = 0.003; HL-RT, *p* = 0.030), hand grip strength (LL-RT, *p* = 0.003; HL-RT, *p* < 0.001), and time of Up and Go test (both *p* < 0.001) following training intervention. All training groups significantly improved lower limb flexibility (AT, *p* = 0.023; LL-RT, *p* < 0.001; HL-RT, *p* < 0.001), upper limb strength (AT, *p* = 0.007; LL-RT, *p* < 0.001; HL-RT, *p* < 0.001), postural balance (AT, *p* = 0.009; LL-RT and HL-RT, both *p* < 0.001), time of STS-5 (AT, *p* = 0.026; LL-RT and HL-RT, both *p* < 0.001), heel raises (AT, *p* = 0.002; LL-RT and HL-RT, both *p* < 0.001), and 6MWT distance (all groups, *p* < 0.001). There was significantly greater improvement in gait speed (+12%, *p* = 0.044), upper limb strength (+13%, *p* = 0.037), Up and Go test time (+9%, *p* < 0.001), and borderline improvement STS-5 time (+9%, *p* = 0.056) in HL-RT group compared with AT group. In addition, Up and Go test time (+18%, *p* < 0.001) and STS-5 time (+14%, *p* = 0.016) were improved significantly more in LL-RT group compared with AT group.

## Discussion

To our knowledge, this is the first study to compare the effects of HL-RT and LL-RT in a combination with AT on body composition and physical performance in patients with CAD. HL-RT and LL-RT decreased only waist circumference and waist-to-hip circumference ratio following training; however, this was not significantly different compared with AT. All training modalities induced favorable effects on flexibility, upper and lower body submaximal muscle strength, and balance. HL-RT improved gait speed and upper body muscle strength to a greater extent compared with AT alone, whereas both HL-RT and LL-RT were associated with greater improvement of STS-5 time.

Previous studies have shown conflicting results of combined AT and RT on anthropometry. One study has shown a similar decrease in waist-to-hip circumference ratio following HL-RT (−0.01) (14) as was observed in our study following HL-RT (−0.01) and LL-RT (−0.02), with no differences when compared with AT. In contrast, the addition of LL-RT to AT failed to induce any changes in waist circumference (13). Similar to our study, previous studies failed to establish the effect of combined AT with RT on body mass (13, 35). Furthermore, combined AT and RT were associated with a decrease in fat mass or fat%(11–13, 35), which was in most studies greater compared with AT (11, 13, 35). On the contrary, we have observed an increase in fat mass% following AT, whereas fat mass% remained unchanged following HL-RT and LL-RT. This discrepancy can be explained by a greater energy requirement needed to maintain body mass while performing RT. In healthy older adults, the energy requirements for engagement in HL-RT increased by nearly 15% (36).

Oppositely to our expectations, HL-RT and LL-RT failed to induce changes in lean body mass and lean body mass%, as reported in the previous studies (11–13, 35). The majority of the available studies on patients with CAD have demonstrated a greater increase in lean body mass following combined RT and AT when compared with AT (11–13, 35). The improvement in lean body mass compared with AT was greater following progressive moderate- to HL-RT (13) or HL-RT (11). In addition, one of the previous studies also showed that a higher weekly frequency of RT (three times vs. once a week) evoked greater gains in lean body mass (13), indicating a potential dose-dependent response of combined RT and AT. In light of previous findings, our results suggest that applying only one resistance exercise (e.g., leg press) in the RT regime failed to provide adequate stimulus for muscle hypertrophy, as was demonstrated in previous studies, which used whole-body resistance exercises (11, 13, 35) and longer RT interventions (24 weeks–1 year) (13, 35). Furthermore, the phase angle was established to be positively associated with physical activity following exercise interventions, while it is still unknown which exercise modality or intensity may provide the greatest benefit (37). Our study showed that neither HL-RT nor LL-RT when combined with AT did not induce favorable changes. At baseline, the phase angle was high; thus, none of the patients were classified as sarcopenic (cut-off: < 4.25° for men and < 4.55° for women) (25). This likely reduced its sensitivity to post-training change in already well-conditioned patients with CAD. Therefore, it seems that this measure is more suitable for frail patients with cardiovascular disease.

The use of physical performance measures remains limited in an age and cardiovascular diagnosis diverse population of patients enrolled in CR (38). Physical performance assessments were mostly used in older patients enrolled in CR (39, 40) and were shown to be safe and feasible (40), with no differences in physical performance levels between minimal (e.g., percutaneous coronary intervention) and more invasive cardiovascular intervention (e.g., coronary artery bypass grafting, aortic valve replacement) (40). In addition to the assessment of maximal aerobic capacity and maximal muscle strength (20), our study was the first to implement an extensive assessment of different physical abilities in middle-aged patients with CAD. Baseline physical performance in our sample of patients was comparable to reference values of similarly aged healthy older adults. For example, the hand grip strength of our patients was between 42 and 43 kg which is in line with reference values of German community-dwelling older adults aged 65 years (men: 42 kg and women: 25 kg) (41). The same observation was found for up and go test, in which our patients even outperformed a healthy population between 60 and 69 years (4.9 s–5.43 s vs. 7.91 s) (42). The distance of 6MWT was above 500 meters which is the general cut-off for a healthy population (43). Our findings, therefore, highlight the importance of the actual physical performance of patients with CAD, which is contrary to the perceptions of most health professionals. Such clinical presumptions may underestimate actual patient performance that reflects in suboptimal exercise loading (during AT and especially RT) resulting in incomplete exploitation of the therapeutic potential of exercise intervention in patients with CAD.

Even though greater gains in physical performance were observed in physically frail patients with cardiovascular disease (39), our well-conditioned patients with CAD improved physical abilities in most of the measured tests, with the exception of gait speed, upper limb flexibility, and Up and Go test time in AT group. We observed a similar increase in gait speed as it was reported following combined AT and very LL-RT in older patients enrolled in CR (39). In addition, our study has also demonstrated that only HL-RT elicited a greater increase in gait speed compared with AT, indicating the importance of RT at higher intensities in previously well-conditioned patients with CAD. Furthermore, our study has shown an improvement in arm curl, STS-5, and heel raises following all three training interventions, which is partially in contrast to other studies (13, 14). Previous two studies in patients with CAD have demonstrated a greater improvement of upper and lower limb muscle endurance and submaximal strength following combined AT and RT when compared to AT, with no post-training changes following AT (13, 14). In our study, the additional improvement in upper and lower muscle strength in AT was potentially modified by our extensive warm-up comprised of dynamic flexibility exercises accompanied by calisthenics using low resistance elastic bands or LL dumbbells. Nevertheless, the improvement in lower limb submaximal muscle strength and mobility (e.g., time of the up and go test, time of STS-5, and heel raises) was greater following HL-RT and LL-RT compared with AT, similarly as reported previously (13, 14). Despite significant improvement in heel raises in all three training groups, only the improvement in LL-RT (+8 reps) and HL-RT (+10 reps) groups were clinically significant, as more than six heel raises were previously established to detect a true change in patients with CAD (44). In addition, the absence of difference between RT groups in outcomes of submaximal muscle strength additionally supports the importance of maximal muscle strength assessment, whereas we showed superior effects of HL-RT over LL-RT (20).

To date, studies that compared the effects of HL-RT and LL-RT on body composition and various physical abilities are limited only to healthy young and older adults (8, 9). While the effects of HL-RT and LL-RT on body composition remain unknown in (un)healthy older adults, a meta-analysis has demonstrated similar effects of both RT modalities on muscle hypertrophy in healthy young adults (9). Such findings were also established in our study; however, the improvement in muscle hypertrophy was lower and non-significant, most likely due to the implementation of only a single lower limb resistance exercise compared with multiple upper and lower limb resistance exercises used in most of the previous interventions (9). Furthermore, another systematic review with meta-analysis comparing HL-RT and LL-RT has shown similar effects of HL-RT and LL-RT on submaximal muscle strength and endurance in healthy older adults (8), as demonstrated in our study. The review also showed no differences in flexibility between intensities, despite improvement in flexibility following both training modalities (8). Moreover, studies in patients with CAD have shown that RT combined with AT did not provide additional improvements in flexibility (11, 15). These findings derived from elderly with and without CAD are in line with our results, and collectively suggest that the flexibility exercises performed usually during warm-up and post-exercise sessions may present an adequate stimulus for flexibility gains, regardless of addition and intensity of RT.

Despite being advised as an adjunct exercise modality (1), the implementation of balance assessments and exercises remains underused in CR, with a scarce body of evidence on the exact characteristics of balance training (39, 45). It seems that the impact of the training intervention is solely related to the duration and complexity of balance training within the CR. In older adults with CAD, the inclusion of few balance exercises failed to promote post-training changes (39), while better structured and progressive balance training implemented in multimodal exercise intervention induced greater improvement in balance test and time of Up and Go test compared with usual care (45). In our study, the addition of complex balance exercises in the warm-up phase of each exercise session enhanced the effects of AT on the Stork balance test in all training groups and has also enhanced greater benefits on time of Up and Go test following HL-RT (+9%) and LL-RT (+18%) compared with AT alone. Furthermore, RT is expected to improve submaximal endurance time or distance in cardiovascular disease patients (17), and most studies implementing RT in their exercise-based CR measured 6MWT distance in elderly patients with CAD (39) or patients with HF (46). Similar to our findings, studies have shown an increase of 6MWT distance following the training intervention, without differences between training modalities (39, 46). Since all three training groups underwent the same progressive AT, our results along with previous studies suggest that AT alone promotes sufficient stimulus for post-training changes in submaximal endurance and that the inclusion of single lower limb RT exercise provides no additional benefits.

Our study has some limitations. To date, no study has compared the differences between HL-RT and LL-RT when combined with AT in patients with CAD; thus, our study was likely underpowered to detect post-training differences between HL-RT and LL-RT in body composition and physical performance. Nevertheless, our study presents one of the largest interventions to compare the effects of combined RT and AT in patients with CAD (5, 6). The assessments of body composition in our study may be limited by the use of bioimpedance, as it is well established that method compared with dual-energy X-ray absorptiometry overestimates lean mass and underestimates fat mass in middle aged to older healthy adults (47). Moreover, the results of our study may also be influenced by the selection of physical performance tests. Despite choosing well-established physical performance tests that were supposed to be most suitable to the age range of patients enrolled in CR (≥65 years) (38), most patients displayed excellent physical performance levels at baseline, which minimized test sensitivity to post-training change. Therefore, future studies should apply maximal assessments of aerobic capacity and muscle strength, regardless of age and conditioning levels of the patients to differentiate the effects of HL-RT and LL-RT. Lastly, the addition of RT was limited only to a single exercise (e.g., leg press machine); thus, the inclusion of upper body, trunk, and calf resistance exercises could yield additional beneficial changes in body composition and whole-body submaximal muscle strength. However, this was not possible due to Coronavirus-19 restriction and absence of medical staff.

## Conclusion

Our study has shown similar beneficial effects of HL-RT and LL-RT when combined with AT on submaximal physical performance during early CR for patients with CAD. Therefore, LL-RT can be used as an alternative to HL-RT for exercise intolerable patients with cardiovascular disease (patients with frailty, sarcopenia, and/or co-existing chronic musculoskeletal syndromes). Still, however, more research is needed to further investigate the feasibility and efficacy of HL-RT over LL-RT using multiple resistance exercises for upper and lower limbs and trunk muscles with balance exercises as an adjunct component. In addition, further research should target to study such effects on older, frail, and/or sarcopenic patients with CAD and heart failure, which would benefit the most from these multimodal interventions in CR.

## Data availability statement

The raw data supporting the conclusions of this article will be made available by the authors, without undue reservation.

## Ethics statement

The studies involving human participants were reviewed and approved by National Medical Ethics Committee of Slovenia (registration number: 0120-573/2019/15). The patients/participants provided their written informed consent to participate in this study.

## Author contributions

TK conceived the study design, recruited and consented the participants to the study, conducted the research, analyzed the data, performed the statistical analysis, interpreted the data, drafted the manuscript, and is responsible for the final content. NŠ conceived the study design and revised the manuscript. ML and VH conceived the study design, supervised the study, revised the manuscript, and are responsible for the final content. All authors approved the final version of the manuscript.

## References

[1] Ambrosetti M Abreu A Corrà U Davos CH Hansen D Frederix I Secondary prevention through comprehensive cardiovascular rehabilitation: from knowledge to implementation. 2020 update. A position paper from the secondary prevention and rehabilitation section of the European Association of Preventive Cardiology. *Eur J Prev Cardiol.* (2021) 28:460–95. 10.1177/2047487320913379 33611446

[2] Visseren FL Mach F Smulders YM Carballo D Koskinas KC Bäck M 2021 ESC Guidelines on cardiovascular disease prevention in clinical practice: Developed by the Task Force for cardiovascular disease prevention in clinical practice with representatives of the European Society of Cardiology and 12 medical societies With the special contribution of the European Association of Preventive Cardiology (EAPC). *Eur Heart J.* (2021) 42, 3227–3337.34458905

[3] Luan X Tian X Zhang H Huang R Li N Chen P Exercise as a prescription for patients with various diseases. *J Sport Health Sci.* (2019) 8:422–41. 31534817 10.1016/j.jshs.2019.04.002 PMC6742679

[4] Vanhees L Geladas N Hansen D Kouidi E Niebauer J Reiner Ž Importance of characteristics and modalities of physical activity and exercise in the management of cardiovascular health in individuals with cardiovascular risk factors: recommendations from the EACPR (Part II). *Eur J Prev Cardiol.* (2011) 19:1005–33. 10.1177/1741826711430926 22637741

[5] Hollings M Mavros Y Freeston J Fiatarone Singh M. The effect of progressive resistance training on aerobic fitness and strength in adults with coronary heart disease: a systematic review and meta-analysis of randomised controlled trials. *Eur J Prev Cardiol.* (2017) 24:1242–59. 10.1177/2047487317713329 28578612

[6] Xanthos PD Gordon BA Kingsley MI. Implementing resistance training in the rehabilitation of coronary heart disease: A systematic review and meta-analysis. *Int J Cardiol.* 230, 493–508. 28040292 10.1016/j.ijcard.2016.12.076

[7] Marzolini S Oh PI Brooks D. Effect of combined aerobic and resistance training versus aerobic training alone in individuals with coronary artery disease: a meta-analysis. *Eur J Prev Cardiol.* (2011) 19:81–94. 21450617 10.1177/1741826710393197

[8] Raymond MJ Bramley-Tzerefos RE Jeffs KJ Winter A Holland AE. Systematic review of high-intensity progressive resistance strength training of the lower limb compared with other intensities of strength training in older adults. *Arch Phys Med Rehabil.* (2013) 94:1458–72. 10.1016/j.apmr.2013.02.022 23473702

[9] Schoenfeld BJ Grgic J Ogborn D Krieger JW. Strength and hypertrophy adaptations between low- versus high-load resistance training. *J Strength Cond Res.* (2017) 31:3508–23. 28834797 10.1519/JSC.0000000000002200

[10] Theodorou AA Panayiotou G Volaklis KA Douda HT Paschalis V Nikolaidis MG Aerobic, resistance and combined training and detraining on body composition, muscle strength, lipid profile and inflammation in coronary artery disease patients. *Res Sport Med.* (2016) 24:171–84. 10.1080/15438627.2016.1191488 27258806

[11] Beniamini Y Rubenstein JJ Faigenbaum AD Lichtenstein AH Crim MC. High-intensity strength training of patients enrolled in an outpatient cardiac rehabilitation program. *J Cardiopulm Rehabil Prev.* (1999) 19:8–17. 10.1097/00008483-199901000-00001 10079415

[12] Pierson LM Herbert WG Norton HJ Kiebzak GM Griffith P Fedor JM Effects of combined aerobic and resistance training versus aerobic training alone in cardiac rehabilitation. *J Cardiopulm Rehabil Prev.* (2001) 21:101–10. 10.1097/00008483-200103000-00007 11314283

[13] Marzolini S Thomas SG Goodman JM. Aerobic and resistance training in coronary disease: single versus multiple sets. *Med Sci Sport Exerc.* (2008) 40:1557–64. 10.1249/MSS.0b013e318177eb7f 18685538

[14] Bäck M Wennerblom B Wittboldt S Cider Å. Effects of high frequency exercise in patients before and after elective percutaneous coronary intervention. *Eur J Cardiovasc Nurs.* (2008) 7:307–13. 18372218 10.1016/j.ejcnurse.2008.02.001

[15] Tofas T Fatouros IG Draganidis D Deli CK Chatzinikolaou A Tziortzis C Effects of cardiovascular, resistance and combined exercise training on cardiovascular, performance and blood redox parameters in coronary artery disease patients: an 8-month training-detraining randomized intervention. *Antioxidants.* (2021) 10:409. 10.3390/antiox10030409 33803076 PMC8001546

[16] Richter D Guasti L Walker D Lambrinou E Lionis C Abreu A Frailty in cardiology: definition, assessment and clinical implications for general cardiology. A consensus document of the Council for Cardiology Practice (CCP), Association for Acute Cardio Vascular Care (ACVC), Association of Cardiovascular Nursing. *Eur J Prev Cardiol.* (2021). 10.1093/eurjpc/zwaa167 34270717

[17] Williams MA Haskell WL Ades PA Amsterdam EA Bittner V Franklin BA Resistance exercise in individuals with and without cardiovascular disease: 2007 update: a scientific statement from the American Heart Association Council on clinical cardiology and council on nutrition, physical activity, and metabolism. *Circulation.* (2007) 116:572–84. 10.1161/CIRCULATIONAHA.107.185214 17638929

[18] Schulz KF Altman DG Moher D. CONSORT 2010 statement: updated guidelines for reporting parallel group randomised trials. *BMJ.* (2010) 340:c332. 10.1136/bmj.c332 PMC2844940 20332509

[19] Kambic T Šarabon N Hadžić V Lainščak M. Effects of high-load and low-load resistance training in patients with coronary artery disease: rationale and design of a randomised controlled clinical trial. *BMJ Open.* (2021) 11:e051325. 10.1136/bmjopen-2021-051325 34301669 PMC8728351

[20] Kambic T Šarabon N Hadžić V Lainscak M. Effects of high- and low-load resistance training in patients with coronary artery disease: A randomized controlled clinical trial. *Eur J Prev Cardiol.* (2022) 4:zwac063. 10.1093/eurjpc/zwac063 35512240

[21] Bjarnason-Wehrens B Mayer-Berger W Meister ER Baum K Hambrecht R Gielen S. Recommendations for resistance exercise in cardiac rehabilitation. Recommendations of the German federation for cardiovascular prevention and rehabilitation. *Eur J Cardiovasc Prev Rehabil.* (2004) 11:352–61. 15292771 10.1097/01.hjr.0000137692.36013.27

[22] Baechle TR Earle RW Wathen D. Resistance training. 3rd ed. In: Baechle TR Earle RW editors. *Essentials of Strength and Conditioning Researchq.* Champagne, IL: Human Kinetics (2008). p. 381–412.

[23] Lamotte M Fleury F Pirard M Jamon A van de Borne P. Acute cardiovascular response to resistance training during cardiac rehabilitation: effect of repetition speed and rest periods. *Eur J Cardiovasc Prev Rehabil.* (2010) 17:329–36. 10.1097/HJR.0b013e328332efdd 20104178

[24] Fletcher GF Ades PA Kligfield P Arena R Balady GJ Bittner VA Exercise standards for testing and training: a scientific statement from the American heart association. *Circulation.* (2013) 128:873–934. 23877260 10.1161/CIR.0b013e31829b5b44

[25] Hirose S Nakajima T Nozawa N Katayanagi S Ishizaka H Mizushima Y Phase angle as an indicator of sarcopenia, malnutrition, and cachexia in inpatients with cardiovascular diseases. *J Clin Med.* (2020) 9:2554. 10.3390/jcm9082554 32781732 PMC7463846

[26] Brzycki M. Strength testing—predicting a one-rep max from reps-to-fatigue. *J Phys Educ Recreat Danc.* (1993) 64:88–90.

[27] Rikli RE Jones CJ. Development and validation of a functional fitness test for community-residing older adults. *J Aging Phys Act.* (1999) 7:129–61. 10.1093/geront/gns071 22613940

[28] Guralnik JM Seeman TE Tinetti ME Nevitt MC Berkman LF. Validation and use of performance measures of functioning in a non-disabled older population: MacArthur studies of successful aging. *Aging Clin Exp Res.* (1994) 6:410–9. 10.1007/BF03324272 7748914

[29] Johnson BL Nelson JK. *The Measurement of Flexibility. Practice Measurement for Evaluation in Physical Education.* Minneapolis, MN: Minnesota Burgess Publishing Company (1979). p. 76–93.

[30] Pierson LM Miller LE Pierson ME Herbert WG Cook JW. Validity of hand-held dynamometry for strength assessment in cardiac rehabilitation. *J Cardiopulm Rehabil Prev.* (2005) 25:266–9. 10.1097/00008483-200509000-00007 16217229

[31] Hébert-Losier K Wessman C Alricsson M Svantesson U. Updated reliability and normative values for the standing heel-rise test in healthy adults. *Physiotherapy.* (2017) 103:446–52. 10.1016/j.physio.2017.03.002 28886865

[32] Hamilton DM Haennel RG. Validity and reliability of the 6-minute walk test in a cardiac rehabilitation population. *J Cardiopulm Rehabil Prev.* (2000) 20:156–64. 10.1097/00008483-200005000-00003 10860197

[33] Borg GA. Psychophysical bases of perceived exertion. *Med Sci Sports Exerc.* (1982) 14:377–81. 7154893

[34] Fatouros IG Kambas A Katrabasas I Nikolaidis K Chatzinikolaou A Leontsini D Strength training and detraining effects on muscular strength, anaerobic power, and mobility of inactive older men are intensity dependent. *Br J Sports Med.* (2005) 39:776L–80L. 10.1136/bjsm.2005.019117 16183776 PMC1725040

[35] Santa-Clara H Fernhall B Mendes M Sardinha L. Effect of a 1 year combined aerobic- and weight-training exercise programme on aerobic capacity and ventilatory threshold in patients suffering from coronary artery disease. *Eur J Appl Physiol.* (2002) 87:568–75. 10.1007/s00421-002-0675-4 12355198

[36] Campbell WW Crim MC Young VR Evans WJ. Increased energy requirements and changes in body composition with resistance training in older adults. *Am J Clin Nutr.* (1994) 60:167–75. 8030593 10.1093/ajcn/60.2.167

[37] Mundstock E Amaral MA Baptista RR Sarria EE Dos Santos RRG Detoni Filho A Association between phase angle from bioelectrical impedance analysis and level of physical activity: systematic review and meta-analysis. *Clin Nutr.* (2019) 38:1504–10. 10.1016/j.clnu.2018.08.031 30224304

[38] Evans J Bethell H Turner S Yadegarfar G. Characteristics of patients entering cardiac rehabilitation in the United Kingdom 1993-2006: implications for the future. *J Cardiopulm Rehabil Prev.* (2011) 31:181–7. 10.1097/HCR.0b013e3181fc0970 21124234

[39] Lutz AH Delligatti A Allsup K Afilalo J Forman DE. Cardiac rehabilitation is associated with improved physical function in frail older adults with cardiovascular disease. *J Cardiopulm Rehabil Prev.* (2020) 40:310–8. 32804797 10.1097/HCR.0000000000000537

[40] Eichler S Hadzic M Völler H Salzwedel A. Octogenarians in interventional cardiology: feasibility and safety of functional and nutritional assessments for a new patient group in cardiac rehabilitation. *Eur J Prev Cardiol.* (2020) 27:2345–7. 10.1177/2047487319899194 32013605

[41] Albrecht BM Stalling I Bammann K. Sex-and age-specific normative values for handgrip strength and components of the senior fitness test in community-dwelling older adults aged 65–75 years in Germany: results from the OUTDOOR ACTIVE study. *BMC Geriatr.* (2021) 21:273. 10.1186/s12877-021-02188-9 33902490 PMC8074447

[42] Long J Cai T Huang X Zhou Y Kuang J Wu L. Reference value for the TUGT in healthy older people: a systematic review and meta-analysis. *Geriatr Nurs.* (2020) 41:325–30. 10.1016/j.gerinurse.2019.11.012 31810729

[43] Dourado VZ Nishiaka RK Simões M Lauria VT Tanni SE Godoy I Classification of cardiorespiratory fitness using the six-minute walk test in adults: comparison with cardiopulmonary exercise testing. *Pulmonology.* (2021) 27:500–8. 10.1016/j.pulmoe.2021.03.006 33958319

[44] Hellmark M Bäck M. Test–retest reliability and responsiveness to change of clinical tests of physical fitness in patients with acute coronary syndrome included in the SWEDEHEART register. *Eur J Cardiovasc Nurs.* (2018) 17:486–95. 10.1177/1474515117743978 29192797

[45] Segev D Hellerstein D Carasso R Dunsky A. The effect of a stability and coordination training programme on balance in older adults with cardiovascular disease: a randomised exploratory study. *Eur J Cardiovasc Nurs.* (2019) 18:736–43. 10.1177/1474515119864201 31328540

[46] Hwang C-L Chien C-L Wu Y-T. Resistance training increases 6-minute walk distance in people with chronic heart failure: a systematic review. *J Physiother.* (2010) 56:87–96. 10.1016/s1836-9553(10)70038-2 20482475

[47] Sillanpää E Cheng S Häkkinen K Finni T Walker S Pesola A Body composition in 18- to 88-year-old adults—comparison of multifrequency bioimpedance and dual-energy X-ray absorptiometry. *Obesity.* (2014) 22:101–9. 10.1002/oby.20583 23894111

